# Rootstock influence on iron uptake responses in *Citrus* leaves and their regulation under the Fe paradox effect

**DOI:** 10.7717/peerj.3553

**Published:** 2017-09-25

**Authors:** Mary-Rus Martinez-Cuenca, Amparo Primo-Capella, Ana Quiñones, Almudena Bermejo, Maria Angeles Forner-Giner

**Affiliations:** 1 Centre of Citriculture and Plant Production, Valencian Agricultural and Research Institute (IVIA), Moncada, Valencia, Spain; 2 Centre of Sustainable Agricultural Development, Valencian Agricultural and Research Institute (IVIA), Moncada, Valencia, Spain

**Keywords:** Fe^2+^, Ferric chelate reductase, Organic acids, Iron transporter, LMWOA, H^+^-ATPase, Apoplast, Fe active, Rootstock

## Abstract

**Background and aims:**

This work evaluates the regulation of iron uptake responses in Citrus leaves and their involvement in the Fe paradox effect.

**Methods:**

Experiments were performed in field-grown ‘Navelina’ trees grafted onto two Cleopatra mandarin × *Poncirus trifoliata* (L.) Raf. hybrids with different Fe-chlorosis symptoms: 030146 (non-chlorotic) and 030122 (chlorotic).

**Results:**

Chlorotic leaves were smaller than non-chlorotic ones for both dry weight (DW) and area basis, and exhibited marked photosynthetic state affection, but reduced catalase and peroxidase enzymatic activities. Although both samples had a similar total Fe concentration on DW, it was lower in chlorotic leaves when expressed on an area basis. A similar pattern was observed for the total Fe concentration in the apoplast and cell sap and in active Fe (Fe^2+^) concentration. *FRO2* gene expression and ferric chelate reductase (FC-R) activity were also lower in chlorotic samples, while *HA1* and *IRT1* were more induced. Despite similar apoplasmic pH, K^+^/Ca^2+^ was higher in chlorotic leaves, and both citrate and malate concentrations in total tissue and apoplast fluid were lower.

**Conclusion:**

(1) The rootstock influences Fe acquisition system in the leaf; (2) the increased sensitivity to Fe-deficiency as revealed by chlorosis and decreased biomass, was correlated with lower FC-R activity and lower organic acid level in leaf cells, which could cause a decreased Fe mobility and trigger other Fe-stress responses in this organ to enhance acidification and Fe uptake inside cells; and (3) the chlorosis paradox phenomenon in citrus likely occurs as a combination of a marked FC-R activity impairment in the leaf and the strong growth inhibition in this organ.

## Introduction

Iron (Fe) is an essential micronutrient for plants as it participates in some life-sustaining processes (Abadía, 1992). Despite it being relatively abundant in many cultivated soils, Fe-deficiency is a common nutritional disorder that causes reduced yield and quality (Álvarez-Fernández et al., 2011) in fruit trees that grow in the Mediterranean basin, where it has been estimated that 20–50% of fruit orchards (including peach, pear, quince, kiwi and citrus) suffer this problem (Abadía et al., 2004; Rombolà & Tagliavini, 2006).

Under Fe-deficient conditions, citrus, like other dicots, have developed the Strategy I mechanism to increase Fe uptake capacity in the root system, which includes increased rhizosphere acidification and Fe^3+^ reduction through proton-ATPases (H^+^-ATPase) and ferric chelate reductase (FC-R) enzymes, respectively, and stimulation of Fe^2+^ transport across root cell membranes mediated by a specific iron-regulated transporter, IRT1 (Vert et al., 2002; Kim & Guerinot, 2007; Ivanov, Brumbarova & Bauer, 2012; Martínez-Cuenca et al., 2013a). In the xylem, Fe^3+^ is transported to leaves chelated by carboxylates (low-molecular-weight organic acids: LMWOA), which accumulates in leaves, xylem and roots in response to Fe-deficiency (López-Millán et al., 2009; Jelali et al., 2010; Martínez-Cuenca et al., 2013b). Transport of the main Fe^3+^ chelate, Fe-citrate, has been linked to a transmembrane protein from the MATE family encoded by the FRD3 gene expressed in the root pericycle and vascular cylinder (Durrett, Gassmann & Rogers, 2007). However, the mechanism that regulates Fe entry into cells at the leaf level, xylem unloading to symplast and re-absorption to apoplast in leaf tissues, has been much less studied than in roots. Although the mechanism that regulates Fe entry into leaf tissue has been much less studied than in roots and it still remains not very clear, ferroportins (FPN1 and FPN2) have been pointed to be involved in Fe vascular loading in the central cylinder and into the vacuole, respectively (Morrissey et al., 2009). While the loss of FPN1 alone is not enough to alter iron sensing, the additional loss of the vacuolar iron buffering of FPN2 adds stress to an impaired system.

According to bibliography, Fe movement in the leaf cells is likely to occur through several responses across the plasma membrane, similar to what happens in the root. Thus, Fe^3+^ compounds have to undergo a second reduction from the leaf apoplast before entering the cell, which is mediated by a PM-bound FC-R enzyme firstly demonstrated in *Vigna unguiculata* by Brüggemann, Maas-Kantel & Moog (1993). Fe^3+^ chelate reductase activities have been detected in leaf disks and protoplasts (De la Guardia & Alcántara, 1996; González-Vallejo et al., 2000; Rombolà et al., 2000; Larbi et al., 2001). In seeds, the Fe reducing activity has been also recently associated to ascorbate efflux that chemically reduce Fe^3+^ from citrate–malate complexes in pea embryos (Grillet, Mari & Schmidt, 2014; Grillet et al., 2014). Shortly afterwards, Fe is transported inside cells by membrane transporter proteins (Nikolic & Römheld, 1999). The *IRT1* gene is expressed in the basal part of flowers, which suggests its role in Fe uptake in aerial tissues in addition to roots (Vert et al., 2002). Some other metal transporters from the ZIP family are also expressed in *Arabidopsis* shoots (Kim & Guerinot, 2007). Fe^2+^ transporters have also been described in organelle membranes, i.e. detected in vitro across the chloroplast inner envelope (Shingles, North & McCarty, 2002).

At this time, it is important to highlight the role of the leaf apoplast in the response to environmental stresses and its function in the transport and storage of several compounds (Dietz, 1997). Regarding Fe, the amount of extra-plasmatic Fe and organic anions that accumulates in the intercellular space could be the cause of genotypical differences in the Fe-deficiency resistance, which is the case in the roots of some plants, including citrus (Longnecker & Welch, 1990; Martínez-Cuenca et al., 2013a). Some authors have related apoplastic pH with Fe mobility and leaf FC-R enzyme activity (Mengel, 1995). Leaf apoplast action on pH, e.g. depressing by spraying acids, has had successful effects on regreening chlorotic leaves (Mengel, 1995; Rombolà et al., 2000). So at this point, apoplast composition is also of crucial importance to understand the processes involved in the Fe uptake mechanism by leaves.

Frequently, Fe-chlorosis disorders on leaves appear due to impaired Fe absorption by the root system and translocation to leaves, and is generated by a short Fe supply due to high levels of bicarbonate, microelements or flooding conditions in soil (Hell & Stephan, 2003; Martínez-Cuenca et al., 2013c, 2013d, 2015). Accordingly, most research available to date about citrus response to Fe-deprived conditions has been conducted in this organ (Chouliaras et al., 2004; Pestana et al., 2005, 2011; Castle, Nunnallee & Manthey, 2009; Martínez-Cuenca et al., 2013a, 2013b, 2015). However, some chlorotic fruit tree leaves on calcareous soils present much higher tissue Fe concentrations than Fe-deficient plants, similarly to plants grown with normal Fe supplies. This phenomenon suggests that chlorosis is not only related to Fe uptake by roots and Fe translocation to upper plant parts, but is also dependent on Fe efficiency in leaves. It has been hypothesised that Fe may be immobilised somewhere in the chlorotic leaf in an unavailable form, which has been termed as ‘Fe-chlorosis paradox’ (Morales et al., 1998; Römheld, 2000). Fe-deficiency works into citrus aerial parts have been restricted to morphological changes in leaves and to evaluating Fe concentration, photosynthetic status, growth or tree production (Maldonado-Torres et al., 2006; González-Mas et al., 2009). As far as we know, regulation of Fe homeostasis at the leaf level in citrus is still unknown.

Therefore, the aim of the present work was to: (1) investigate the regulation of the Fe uptake system by leaf symplasm in citrus; (2) determine the role of apoplast composition in Fe homeostasis in leaves; and (3) evaluate the effect of two different citrus rootstocks (Cleopatra mandarin × *Poncirus trifoliate*; both hybrids from mother plants that were tolerant and sensitive to Fe-chlorosis, respectively (Castle, Nunnallee & Manthey, 2009) on the similar total Fe concentration found in leaves of ‘Navelina’ trees grown at field Fe-limiting conditions (high HCO_3_^−^ concentration in soil). For this purpose, physiological responses (chlorophyll concentration, photosynthesis rate, peroxidase and catalase activities) and total Fe and Fe^2+^ form concentrations in leaves were determined. The expression of some genes that encode for the enzymes involved in strategy I responses in the root was also examined in leaves. The LMWOA concentration in leaves and pH in the apoplast extract were also measured.

## Materials and Methods

### Plant material and growth conditions

Our experiment was run in an experimental orchard in the town of Moncada (Valencia) Spain, 38°14′53.57″N; 0°41′46.9″W), which is a subarid region in the Turia River upper basin. The annual average values of the study site are 17.3 °C, 390.1 mm precipitation and 65.8% air humidity (IVIA). Sandy loam soil was the classification for the soil texture within the first 50 cm depth. pH 8.5, 1.63% organic matter (w/w), 40% total calcium carbonate (w/w) and 8% deactive calcium carbonate were the main chemical soil characteristics. A 5–10% elative level of carbonate is considered high.

Measurements were obtained from the leaves of 18-year-old ‘Navelina’ orange trees, *Citrus sinensis* (L.) Osb. (selection Iniasel 7), which has been grafted onto two citrus rootstocks taken from Cleopatra mandarin × *Poncirus trifoliata* (L.) Raf. mother plants (J. Forner, IVIA) in as citrus rootstocks breeding programme to assess the new citrus rootstocks’ behaviour when faced with several abiotic disorders. The selected material displayed distinct Fe-chlorosis symptoms: 030146 (non-chlorotic) and 030122 (chlorotic).

In 1993, trees (2.5 × 6 m) were planted with nursery plants (12 months post-grafting). They were arranged six trees per rootstock in a completely randomised experimental plot. Another row of guard trees separated the experimental blocks from one another. Standard cultural practices included fertilising and drip irrigation, mechanical inter-row weed control and inter-tree chemical control. Drip irrigation frequency was modified to seasons, and ranged from once weekly (winter) to five days/week (summer), with 40 L tree^−1^ per irrigation. Electrical conductivity was 1.4 dS m^−1^, Cl^−^ 134.8 mg L^−1^ and NO_3_^−^, and water pH was 7.6. Determinations were recorded in spring flush fully expanded leaves taken from the non-fruiting shoots collected in October 2014.

### Leaf growth measurements

Random sampling was done using 10 leaves per tree from each tree’s canopy periphery. Leaves were rinsed under a tap, and also with distilled water that contained a non-ionic detergent, and were finally rinsed three times with distilled water. An LI-300 area-meter (LI-COR, Lincoln, NE, USA) was used to measure total leaf area with and a forced draft oven was employed to determine biomass after drying for 48 h at 70 °C to achieve constant dry weight (DW, in g). Measurements are presented as the mean ± SE of the six replicates per scion:rootstock combination. The average value of six technical replicates was taken as being representative of each individual plant.

### Chlorophyll concentration and gas exchange parameters

The leaf chlorophyll (Chl) concentration was spectrophotometrically measured (Lambda 25; PerkinElmer, Shelton, CT, USA), as described by Moran & Porath (1980). Leaf discs (Ø = 7 mm; no major veins) were acquired with a cork borer from spring leaves. The samples with 0.06 g of fresh weight (four leaves gave 12 discs) were incubated in 6 mL of *n*,*n*-dimethylformamide for 24 h at 4 °C, and were then centrifuged at 6,000*g* and 4 °C for 15 min. The supernatant was left for 60 min in the presence of Na_2_SO_4_ and absorbance was measured at 664 and 647 nm (Lambda 25; PerkinElmer, Shelton, CT, USA). The Chl concentration was estimated non-destructively in a SPAD portable apparatus (Minolta Co., Osaka, Japan), and the method was previously calibrated according to Abadía & Abadía (1993).

A portable chlorophyll fluorometer (PAM-2100; Heinz Walz Gmbh, Germany) was employed to record the chlorophyll fluorescence parameters (*F*_o_: minimal fluorescence, *F*_m_: maximal fluorescence and *F*_v_ = *F*_m_−*F*_o_: variable fluorescence). After a 30 min dark adaptation period, a saturating pulse of 2,100 μmol quanta m^−2^ s^−1^ for 5 s leaves was used for illuminating purposes, and *F*_o_ and *F*_m_ measurements were taken.

The photosynthetic activity (net CO_2_ assimilation rate, A_CO2_) of the single attached leaves was measured between 10:00 and 11:30 h on a sunny day so that measurements could be taken when conditions were relatively stable. Photosynthetically active radiation on was adjusted the leaf surface to a photon flux density of 1,000 μmol m^−2^ s^−1^. Closed gas exchange (CIRAS-2; PP-systems, Hitchin, UK) was used to take measurements. Leaf laminae were completely enclosed in a PLC 6 (U) universal leaf autocuvette inside a closed circuit model, and were left at 25 ± 0.5 °C, with a leaf-to-air vapour deficit of approximately 1.7 Pa. Through the cuvette, the air flow rate was 500–1,500 mL min^−1^. Three-second intervals were used to take 10 consecutive measurements.

Measurements on spring fully expanded leaves were taken and are the mean ± SE of the six replicates per scion:rootstock combination. The average value of three technical replicates (four leaves each) for the Chl data was considered to be representative of each individual plant. The average value of six leaves for the *F*_v_/*F*_m_ and A_CO2_ parameters was taken as being representative of each individual plant.

### Catalase and peroxidase activities

Catalase (CAT) and peroxidase (POX) enzyme activities were determined according to Forner-Giner et al. (2010). A Polytron 3100 (Kinematica, Switzerland) was used homogenise fresh sample (2 g) in 10 mL of 10 mM phosphate buffer (pH 6.5) and 2.5% (w/v) insoluble polyvinylpolypyrrolidone (PVPP). The crude extract was then centrifuged for 30 min at 12,000 rpm and 4 °C. The supernatant was collected and utilised to determine soluble POX and CAT activities. Enzyme activities were analysed by measuring the reduction in hydrogen peroxide (H_2_O_2_), and were spectrophotometrically determined (Lambda 25; PerkinElmer, Shelton, CT, USA) and were taken as fresh weight (U g^−1^ FW). The molar extinction coefficient was 43.6 M^−1^ cm^−1^.

Adding 10 mM H_2_O_2_ commenced CAT activity (final volume of 2 mL) in a reaction mixture with 50 mM of phosphate buffer (pH 7.0) and 100 μL of supernatant. Reaction monitoring was done for 5 min at 240 nm and room temperature. POX activity (final volume of 2 mL) was established in a reaction mixture. This mixture comprised 50 mM of glycine buffer (pH 4.5), 0.8 mM of H_2_O_2_, 50 mM of ABTS [2,2-azino-bis (3-ethylbenzothiazoline-6-sulphonic acid)] and 100 μL of enzyme extract. Any change in absorbance was monitored for 5 min at 590 nm and room temperature.

Measurements are presented as the mean ± SE of the six replicates per scion:rootstock combination. The average value of three technical replicates was taken as representing each individual plant.

### Cell fluid isolation

Fresh leaves were harvested by using a razor blade to cut petioles at the leaf lamina base. After xylem sap removal, apoplasmic fluid was collected as the methodology described by Dannel, Pfeffer & Marschner (1995) sets out, but with a few modifications. These procedures were followed at 4 °C to minimise apoplasmic fluid composition changes. Leaves (about 10 g of FW) were placed so that all the cut lay in the same direction, were tightened into a roll with plastic foil, placed in a 10 mL syringe and were centrifuged. Eliminating the fluid collected at an initial 1,500*g* force avoided cytosolic contamination. Apoplasmic fluid was collected after the subsequent centrifugal force for 15 min at 4,500*g* and 4 °C (Eppendorf Centrifuge 5810R; Eppendorf AG, Hamburg, Germany) and by filtering through a 0.20 μm polyvinyl fluoride (PVF) syringe filters (OlimPeak; Teknokroma, Sant Cugat del Vallés, Spain). Apoplasmic fluid was divided into two subsamples; one was divided into 0.5 mL aliquots, frozen in liquid N_2_ and stored at −80 °C until used; the other was employed to take pH measurements (Jenway 3520 Stone; Jenway, Staffordshire, UK). The three apoplasmic fluids combination was considered to represent each individual plant.

Cell sap from centrifuged leaves was collected by freezing in liquid N_2_ to rupture cells, storing at −18 °C for approximately 24 h and using a hydraulic press to press leaves after they had been thawed. Cell sap was divided into 1 mL aliquots, frozen in liquid N_2_ and stored at −80 °C until used. The three cell sap extractions combination was considered representative of each individual plant.

### Total ion extractions

Thirty leaves per tree canopy periphery were randomly sampled to be independently pooled and analysed. Leaves were firstly rinsed with distilled de-ionised water. Then they were frozen in liquid N_2_, freeze-dried (Telstar LyoAlfa 6; Terrassa, Barcelona, Spain) and crushed in a hammer-mill. Finally, they were stored at room temperature and in the dark until further analyses were run.

The total Fe, K and Ca concentrations in whole leaves were analysed from 0.5 g DW of the leaf tissue burnt for 12 h in a muffle furnace at 550 °C, extracted with 2% nitric acid (Hiperpur Panreac, Fe <1 ppb) in an ultrasonic bath for 30 min at 40 °C (Fungilab S.A., Sant Feliu de Llobregat, Barcelona, Spain) and diluted to 25 mL with ultrapure water. The total Fe concentration in the apoplasmic and cell sap fluids was analysed from 0.5 mL of the extract, which was freeze-dried and treated as formerly described for DW material. Final preparations were stored at 4 °C until used. An AAS model Perkin Elmer Analyst (Waltham, Millipore, Molsheim, France, MA, USA) was used to measure ion concentrations. Ultra-pure water (Ultra Pure Water Systems Milli Q Plus; Millipore, Billerica, MA, USA) was employed in all the analyses. Measurements are presented as the mean ± SE of the six replicates per scion:rootstock combination.

### Ferrous analysis

The ferrous concentration in both apoplasmic fluid and cell sap was established by the *o*-phenanthroline extraction method (Katyal & Sharma, 1980, but with some modifications; Manzanares, Lucena & Garate, 1990; Nikolic & Kastori, 2000; Başar, 2003). Fluid extracts (0.5 mL) were placed inside 10 mL pyrex tubes and Fe^2+^ ions were extracted using 2.5 mL of 1.5% *o*-phenanthroline in HCl-buffer (pH 3.0). Samples were gently homogenised by vortexing and were left to stand at room temperature for 16 h. Extracts were centrifuged for 5 min at 1,000 rpm and Whatman No. 1 filter paper was used to filter the supernatant. The filtrate’s ferrous (Fe^2+^) content was established by spectrophotometry at the 510 nm wavelength (Lambda 25; PerkinElmer, Shelton, CT, USA). Measurements are presented as the mean ± SE of the six replicates per scion:rootstock combination.

### RNA extraction and real-time RT-PCR analysis

Five leaves were randomly sampled per tree canopy periphery and were then washed. Major veins were eliminated and ground in a mortar in N_2_. Total leaf tissue RNA (0.1 g) extraction was performed with the RNeasy Plant Mini Kit (Qiagen, Hilden, Germany). RNA samples were treated with RNase-free DNase (Qiagen) by column purification, following the manufacturer’s instructions, to remove genomic DNA. RNA quality (OD_260_/OD_280_ ratio) and concentration were evaluated in an ND-1000 full spectrum UV–Vis spectrophotometer (Nanodrop Technologies, Thermo Fisher Scientific, Wilmington, DE, USA). A quantitative real-time reverse transcription polymerase chain reaction (RT-PCR) was run in a LightCycler 2.0 Instrument (Roche, Mannheim, Germany), equipped with version 4.0 of the Light Cycler Software. Reactions contained the following: 2.5 units of MultiScribe Reverse Transcriptase (Applied Biosystems; Roche Molecular Systems, Branchburg, NJ, USA); 1 unit of RNase Inhibitor (Applied Biosystems); 2 μL of LC Fast Start DNA Master PLUS SYBR Green I (Roche Diagnostics GmbH, Mannheim, Germany); 25 ng of total RNA; 0.250 μM of the specific forward and reverse primers of each gene in a total 10 μL volume. Incubations lasted 30 min at 48 °C, followed by, 95 °C for 10 min, and then by 45 cycles at 95 °C for 2 s, 58 °C for 8 s and 72 °C for 8 s. The fluorescent intensity data were obtained in the 72 °C extension step. They were transformed into relative mRNA values with a 10-fold dilution series of an RNA sample taken as the standard curve. The relative mRNA levels were normalised to total RNA amounts, as formerly described (Bustin, 2002; Hashimoto, Beadles-Bohling & Wiren, 2004) and the non-chlorotic sample values were arbitrarily assigned an expression value of 1. The reference gene was actin (Yan et al., 2012). The amplification reactions’ specificity was evaluated by post-amplification dissociation curves and by reaction product sequencing. The resolutions of the curve expressions were confirmed.

A homology search was run with the related genes from the ‘Haploid Clementine Genome, International Citrus Genome Consortium (Goodstein et al., 2012; http://www.phytozome.net/clementine)’ to identify putative genes. Synthetic oligonucleotides were designed to amplify the gene from selected clones, and were sequenced for confirmation as previously mentioned. Table 1 provides details of the forward and reverse primers used. Measurements are the mean ± SE of the six replicates per scion:rootstock combination. At least three RT-PCR reactions were performed with three technical replicates per sample.

**Table 1:** List of primers used for quantitative real-time PCR.

Annotation	Code^a^	Forward/reverse primer (5′–3′)	Predicted product (bp)
*FRO2*	Clementine0.9_000943m	GGAGGAGCCAAAACAAGATG	73
		CAGCCAAGAAACACAGCAAA	
*HA1*	Clementine0.9_001614m	GGACGCGTTTGGTGTAAGAT	143
		GAAGTCCAGGGCGTTCAATA	
*IRT1*	Clementine0.9_014282m	CTCAGTTGGAGCCACAAACA	115
		GTACTCCGCCTGAAGAATGC	
*ACT*	Clementine0.9_013166m	TTAACCCCAAGGCCAACAGA	141
		TCCCTCATAGATTGGTACAGTATGAGAC	

**Note:**

a Code refers to the transcript name in the database available in the International Citrus Genome Consortium (ICGC; http://www.phytozome.net/search.php).

### Fe chelate reductase (FC-R) activity

Ferric chelate reductase activity was established by measuring the formation of Fe^2+^ and the BPDS (bathophenanthroline-disulfonic acid disodium salt hydrate) complex from FeEDTA (De la Guardia & Alcántara, 1996). Leaf discs without major veins (Ø = 7 mm) were obtained from spring leaves using a cork borer. The samples that contained 0.1 g FW (20 discs from five leaves) were placed inside 10 mL glass test tubes and were washed in 5 mL of 0.5 mM CaSO_4_ for 30 min. Then, they were incubated in 5 mL of assay medium (0.5 mM of CaSO_4_, 1 mM of KCl, 50 mM of potassium phosphate buffer, pH 7.0, 0.1 mM of FeEDTA and 0.3 mM of BPDS). Rubber stoppers were used to seal tubes, which were completely covered with aluminium foil to avoid light entering. Blanks with no leaf disks were run under the same conditions with FeEDTA and in the presence of BPDS to calculate the degree of photochemical reduction. The control tubes with the plant material, but with no Fe source, were included to evaluate the release of reducing compounds by leaf disks (Larbi et al., 2001). Incubation was run at 23 °C for 24 h in the darkness. Absorbance was determined at 535 nm by spectrophotometry (Lambda 25; PerkinElmer, Shelton, CT, USA). BPDS formed a stable water-soluble red complex with Fe^2+^, and only a weak complex with Fe^3+^. The quantity of reduced Fe was calculated by the Fe^2+^-(BPDS)_3_ complex concentration with an extinction coefficient of 22.14 mM^−1^ cm^−1^. Finally, it was expressed as nmol Fe^2+^ reduced g^−1^ leaf FW h^−1^.

Measurements are the mean ± SE of the six replicates per scion:rootstock combination. The average value of the three technical replicates was taken to represent each individual plant.

### Extraction and determination of LMWOAs

Total LMWOAs were extracted in accordance with Veberic et al. (2010), but with some modifications. Leaf samples (0.05 g DW) were homogenised (Kinematica Polytron PT 3100; Kinematica, Lucerne, Switzerland) at 15,000*g* for 30 min in 2 mL of H_2_SO_4_ 0.1%, and were then centrifuged for 30 min at 7,500*g* and 4 °C (Eppendorf Centrifuge 5810R; Eppendorf AG, Hamburg, Germany). The supernatant was collected and filtered through a 0.45 μm PVF syringe filter (OlimPeak; Teknokroma, Sant Cugat del Vallés, Spain). Final extracts were divided into 0.6 mL aliquots, frozen in liquid N_2_ and stored at −80 °C until used.

The LMWOAs (malate and citrate) in both the total leaf and apoplast fluid were established by HPLC in an Alliance liquid chromatographic system (Waters, Barcelona, Spain), equipped with a 2,695 separation module coupled to a 2,996 photodiode array detector and a ZQ2000 mass detector. Data acquisition was performed with the Empower 2 software (Waters, Barcelona, Spain). Sample temperature and column temperature were 5 and 35 °C, respectively. Other values were: capillary voltage, 3.0 kV; cone voltage, 23 V; source temperature, 100 °C; desolvation temperature, 200 °C; desolvation gas flow, 400 L h^–1^. Full data acquisition was done using electrospray ion negative conditions by scanning from 100 to 400 uma in the centroid mode. Chromatographic separation involved the use of an ICSep ICE-COREGEL 87H3 column (Transgenomic, Glasgow, UK), an ICSep ICE-COREGEL 87H guard kit and an automatic injector. An isocratic mobile phase of 0.008 N H_2_SO_4_ solution was the solvent system. The total run time at 0.6 mL min^–1^ was 20 min, and the injection volume was 5 μL. Compounds were identified by comparing their retention times, UV–Vis spectra and mass spectrum data to the corresponding authentic standards. Standards were run daily with the samples utilised for validation. Concentrations were established by an external calibration curve with citric and acid malic acid, purchased from Sigma (Sigma Co., Barcelona, Spain). Solvents and water were of HPLC-MS grade.

### Statistical analyses

The obtained data underwent an ANOVA. Data distributions were checked for normality and statistical analyses were done by ANOVA with version 5.1 of Statgraphics Plus (Statistical Graphics, Englewood Cliffs, NJ, USA). The mean values were compared at the 95% confidence level by the least significant differences (LSD) method.

## Results

### Fe-deficiency symptoms in leaves

**Figure 1:**
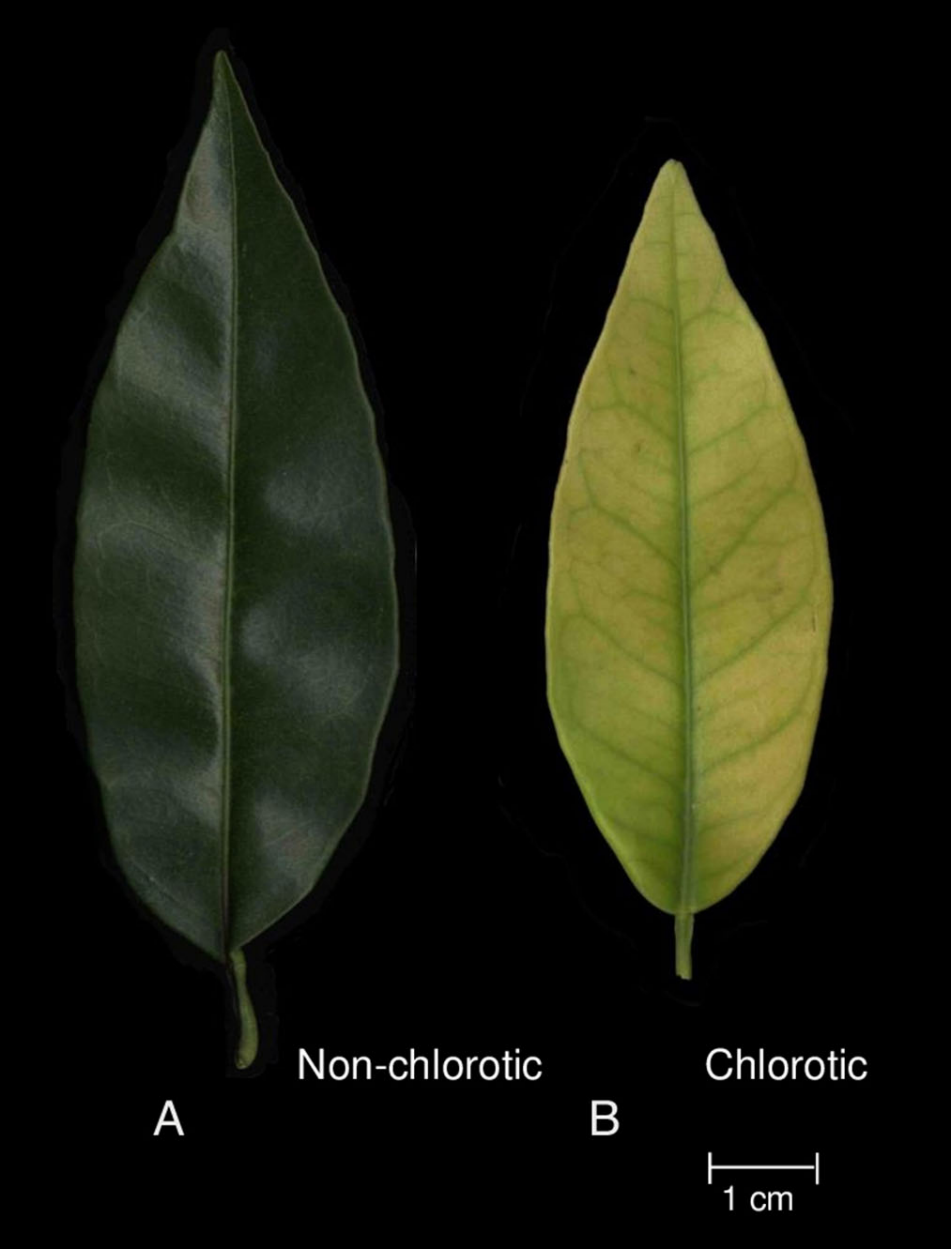
Visual symptoms of Fe-chlorosis in the fully expanded leaves of ‘Navelina’ orange trees grafted on (A) non-chlorotic (030146) and (B) chlorotic (030122) rootstocks.

The fully expanded leaves of the ‘Navelina’ orange trees grafted onto rootstocks 030146 and 030122 displayed striking differences in the non-chlorotic and chlorotic Fe-chlorosis symptoms, respectively (Fig. 1), and some leaf growth parameters (Table 2). The samples that exhibited Fe-deficiency symptoms were smaller than the non-chlorotic leaves on both DW and area bases (36.6% and 26.6%, respectively).

**Table 2:** Leaf growth parameters (biomass, in g DW; and leaf expansion area, in cm^2^) and total Fe concentration on DW (in μg g^−1^DW) and surface (in μg cm^−2^) bases measured in the non-chlorotic and chlorotic fully expanded leaves of ‘Navelina’ orange trees.

	Non-chlorotic	Chlorotic	ANOVA
Leaf growth parameters			
Biomass (g DW)	0.358	0.227	^*^
Leaf expansion area (cm^2^)	21.4	15.7	^*^
Total Fe concentration			
μg g^−1^ DW	65.6	60.7	ns
μg cm^−2^	1.10	0.88	^*^

**Notes:**

Values are the mean of six biological replicates (*n* = 6). For a comparison of mean, a variance analysis (ANOVA), followed by the least significant differences (LSD) test, calculated at the 95% confidence level, was performed. Significant differences are indicated as follows:

* *p* < 0.05; ns, not significant; DW, dry weight.

### Total Fe and Fe^2+^ concentrations

The Fe concentration in the total leaf tissue in both samples was similar when referenced to biomass (about 63 μg g^−1^ of DW; Table 2). This parameter differed significantly between both chlorotic states in relation to leaf area. The non-chlorotic leaves had an Fe total concentration of 1.10 μg Fe cm^−2^, and this value lowered by 20% when analysed in the chlorotic samples.

**Figure 2:**
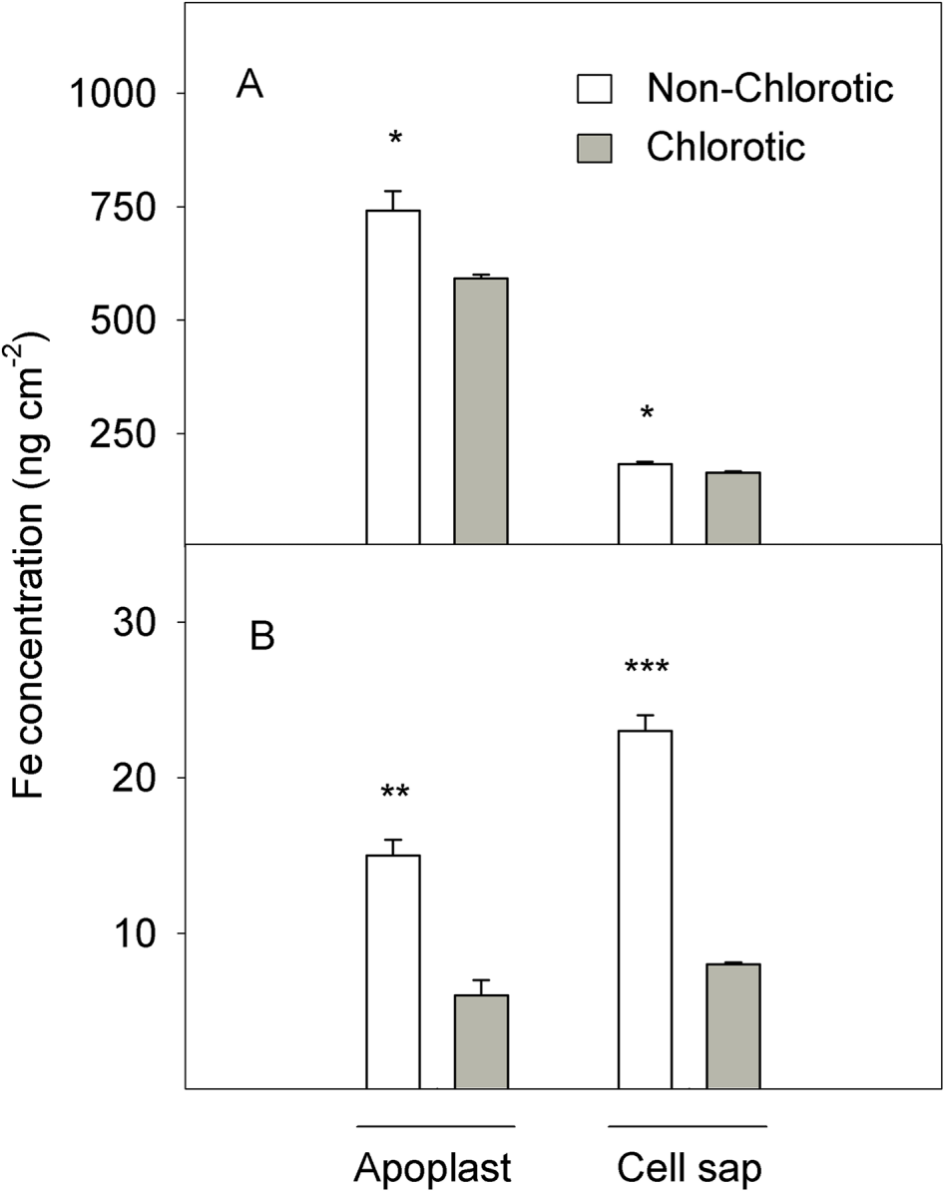
(A) Total Fe and (B) Fe^2+^ concentrations (in ng cm^−2^) measured in the apoplast and cell sap extracts of the non-chlorotic and chlorotic fully expanded leaves of ‘Navelina’ orange trees. Values are the mean ± SE of six biological replicates (*n* = 6). For a comparison of mean, a variance analysis (ANOVA), followed by the least significant differences (LSD) test, calculated at the 95% confidence level, was performed. Significant differences are indicated as follows: *p < 0.05; **p < 0.01; ***p < 0.001.

A similar total tissue pattern was maintained when analysing the Fe concentration in the apoplast and cell sap fluids (Fig. 2). On the area basis, both leaf types accumulated more total Fe in the leaf apoplast than in cell sap (4.1- and 3.6-fold respectively for non-chlorotic and chlorotic leaves). However, significant differences between the samples with different chlorotic symptoms were observed. The Fe concentration in the chlorotic leaves was respectively 20.3% and 10.3% lower in the apoplast and cell sap than in the non-chlorotic leaves.

Figure 2 illustrates the Fe accumulation in the active form (Fe^2+^) in the apoplast and cell sap extracts of leaf tissue (expressed on the area basis). In quantitative terms, the quantity of Fe^2+^ was substantially lower than total Fe, but marked differences between samples appeared. The non-chlorotic leaves had 2.5- and 2.8-fold higher Fe^2+^ concentrations than the chlorotic ones. The main Fe^2+^ accumulation in leaves with no Fe-deficiency symptoms was observed in the cell sap extract.

### Photosynthetic parameters and antioxidant activities

Chlorotic leaves displayed marked reductions in *Chl a* and *b* concentrations (60.7% and 48.2%, respectively) versus the non-chlorotic samples (Table 3), which corresponded to a lowering of 24.8% in the Chl a/b ratio for the former. The SPAD value in the chlorotic leaves was also lower (62.1% decrease) than in green leaves. According to these results, Fe-deficiency symptoms caused major changes to the chlorophyll fluorescence parameters versus with the non-chlorotic leaves, and the maximum quantum PSII yield lowered by 18.1% (*F*_v_/*F*_m_; Table 3). The net photosynthetic rate (A_CO2_) lowered by 16.4% in the chlorotic leaves versus the non-chlorotic ones (Table 3).

**Table 3:** Chlorophyll (Chl) concentration (in μmol g^−1^ DW), SPAD index, fluorescence (*F*_v_/*F*_m_) parameter and photosynthetic rate (A_CO2_, in μmol m^−2^ s^−1^) measured in the non-chlorotic and chlorotic fully expanded leaves of ‘Navelina’ orange trees.

	Non-chlorotic	Chlorotic	ANOVA
*Chl a*	3.6	1.4	^***^
*Chl b*	1.4	0.7	^**^
*Chl a/b*	2.6	1.9	^*^
SPAD	70.2	26.6	^***^
*F*_v_/*F*_m_	0.795	0.651	^*^
A_CO2_	7.2	5.3	^**^

**Notes:**

Values are the mean of six biological replicates (*n* = 6). For the comparison of mean, a variance analysis (ANOVA), followed by the least significant differences (LSD) test, calculated at the 95% confidence level, was performed. Significant differences are indicated as follows:

* *p* < 0.05;

** *p* < 0.01;

*** *p* < 0.001. DW, dry weight.

**Figure 3:**
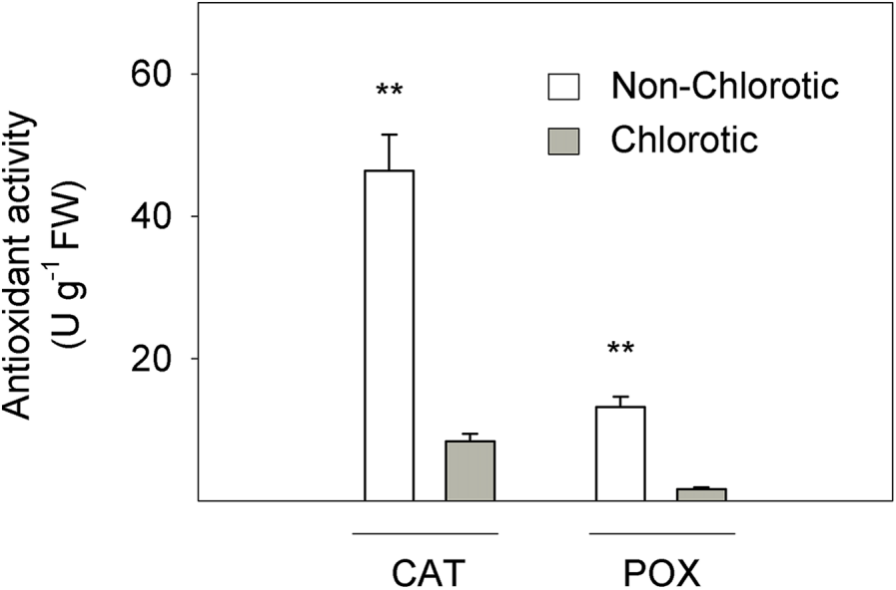
Catalase (CAT) and peroxidase (POX) activities (in U g^−1^ FW) measured in the non-chlorotic and chlorotic fully expanded leaves of ‘Navelina’ orange trees. Values are the mean ± SE of six biological replicates (*n* = 6). For a comparison of mean, a variance analysis (ANOVA), followed by the least significant differences (LSD) test, calculated at the 95% confidence level, was performed. Significant differences are indicated as follows: **p < 0.01. FW, fresh weight.

Figure 3 depicts the activity of CAT and POX enzymes in relation to the antioxidant response to Fe-chlorosis in leaves. CAT and POX enzyme activities were respectively 5.5- and 7.8-fold higher in the non-chlorotic leaves than in the chlorotic ones.

### Fe uptake responses in leaves

**Figure 4:**
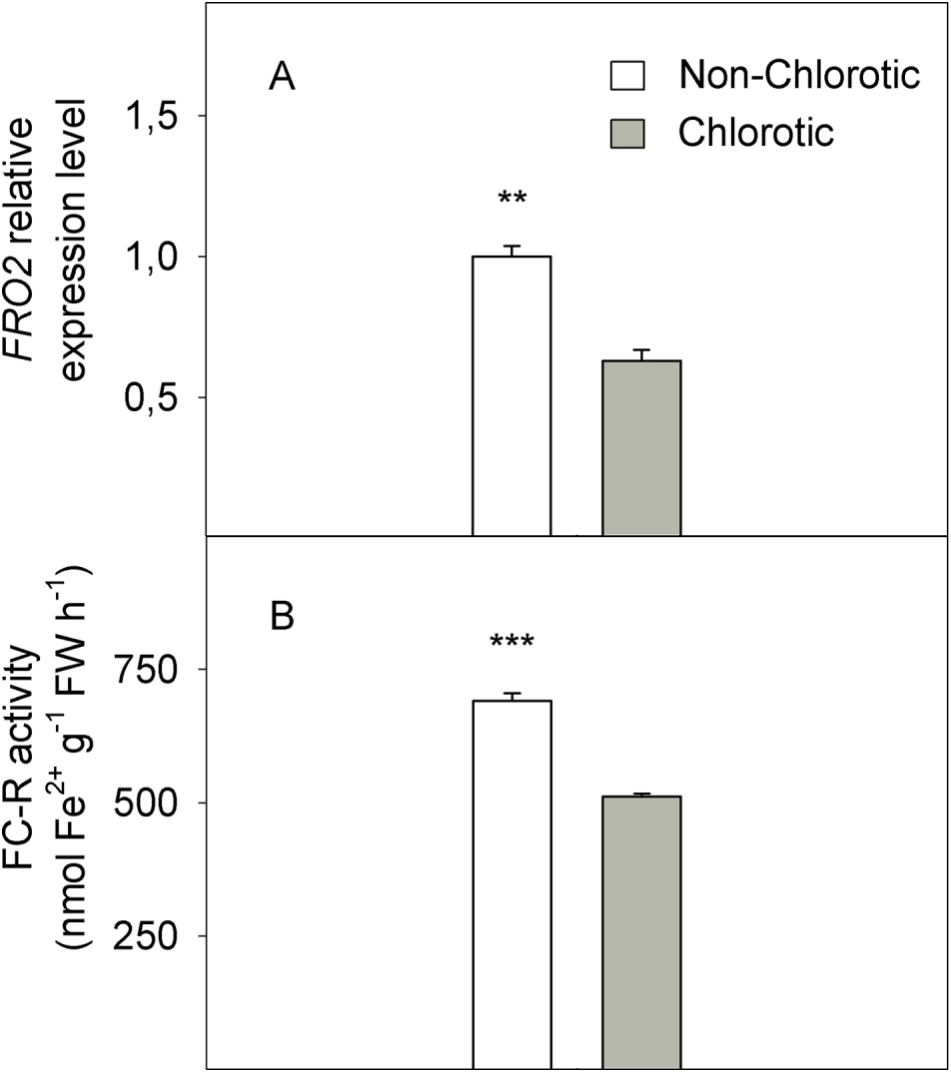
(A) Relative expression of the *FRO2* gene determined by real-time RT-PCR analysis and (B) ferric-chelate reductase (FC-R) activity (in nmol Fe^2+^ g^−1^ FW h^−1^) measured in the non-chlorotic and chlorotic fully expanded leaves of ‘Navelina’ orange trees. Values are the mean ± SE of six biological replicates (*n* = 6). For a comparison of mean, a variance analysis (ANOVA), followed by the least significant differences (LSD) test, calculated at the 95% confidence level, was performed. Significant differences are indicated as follows: **p < 0.01; ***p < 0.001. FW, fresh weight.

In molecular terms, the expression level of FC-R gene *FRO2* dropped by 41% in the chlorotic leaves versus the non-chlorotic ones (Fig. 4). The activity of this enzyme strongly paralleled gene behaviour and was 1.3-fold lower in the leaves with chlorosis symptoms than in those without them.

**Figure 5:**
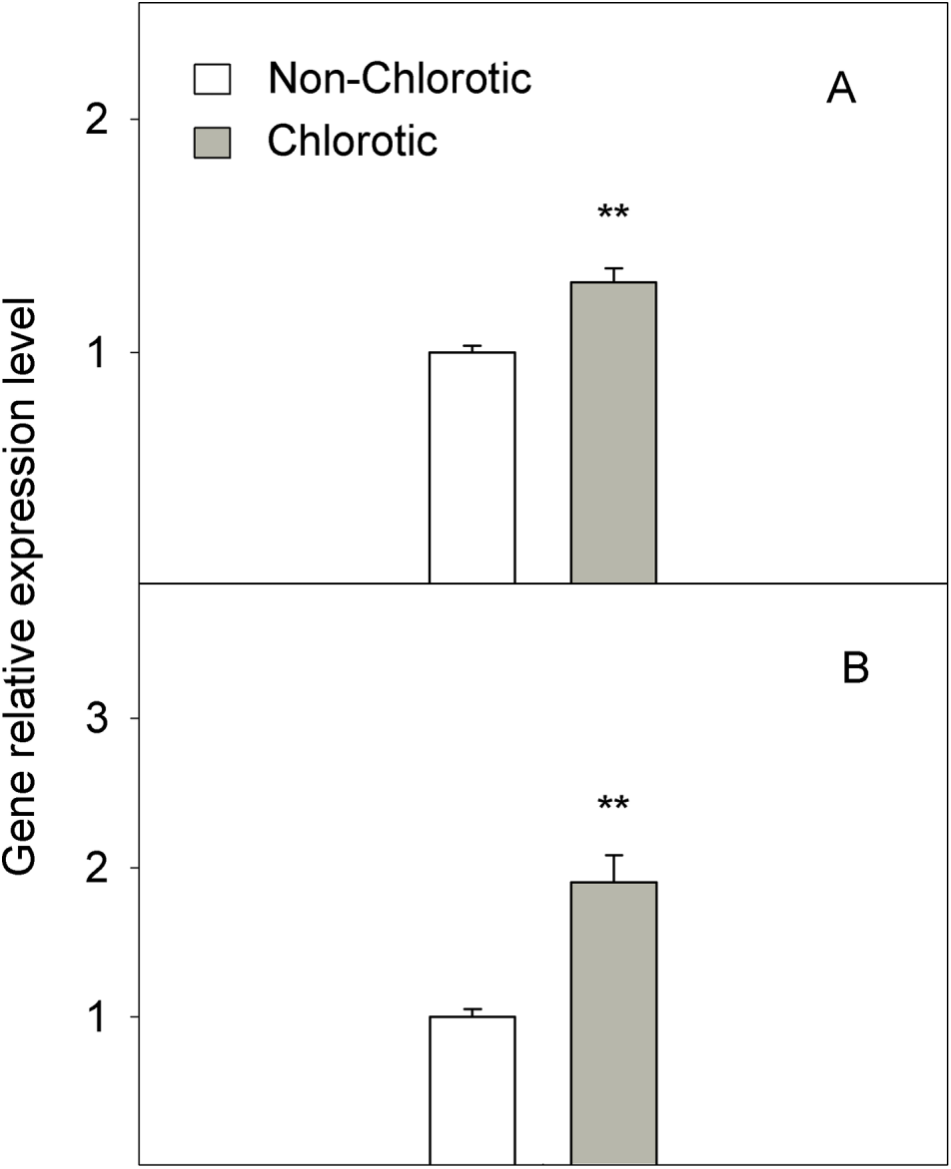
Relative expression of genes (A) *HA1* and (B) *IRT1* determined by a real-time RT-PCR analysis measured in the non-chlorotic and chlorotic fully expanded leaves of ‘Navelina’ orange trees. Values are the mean ± SE of six biological replicates (*n* = 6). For a comparison of mean, a variance analysis (ANOVA), followed by the least significant differences (LSD) test, calculated at the 95% confidence level, was performed. Significant differences are indicated as follows: **p < 0.01.

**Figure 6:**
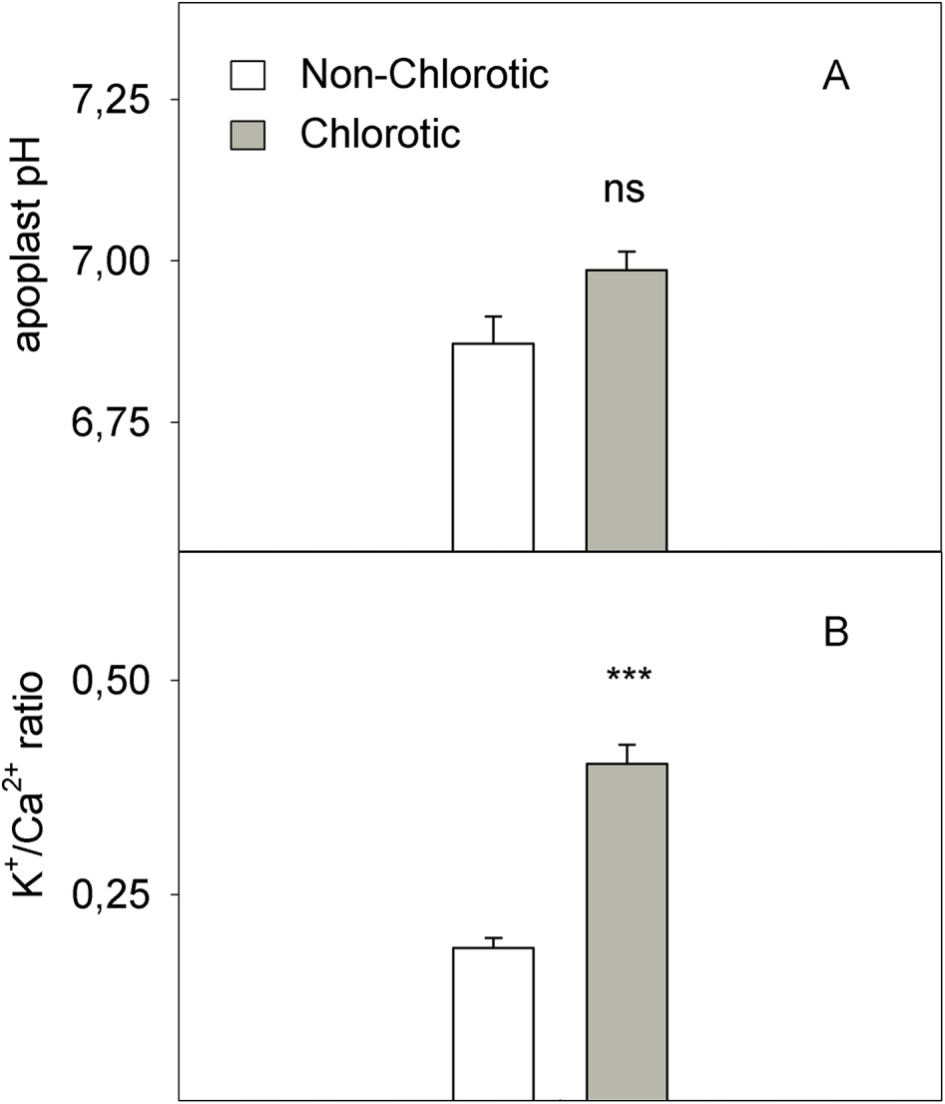
(A) pH in leaf apoplastic fluid and (B) the K^+^/Ca^2+^ ratio in whole leaf measured in the non-chlorotic and chlorotic fully expanded leaves of ‘Navelina’ orange trees. Values are the mean ± SE of six biological replicates (*n* = 6). For a comparison of mean, a variance analysis (ANOVA), followed by the least significant differences (LSD) test, calculated at the 95% confidence level, was performed. Significant differences are indicated as follows: ***p < 0.001; ns, not significant.

The chlorotic leaves showed increases of 31.8% and 94.2% of mRNA transcripts abundance respectively for the H^+^-ATPase *HA1* and iron transporter *IRT1* genes (Fig. 5). The proton extrusion between the leaf symplast and apoplast, established by the pH measurements in leaf apoplast fluid, gave values of 6.87 and 6.98 in the non-chlorotic and chlorotic leaves, respectively (Fig. 6). The results were not significantly different despite the apparently lower pH in the former. The ratio between the K^+^ and Ca^2+^ concentrations (K^+^/Ca^2+^) in whole leaves, in relation to H^+^ excretion across the plasma membrane, was 2.2-fold higher in the chlorotic leaves than in those samples that lacked Fe-chlorosis symptoms (Fig. 6).

### LMWOA concentrations

**Figure 7:**
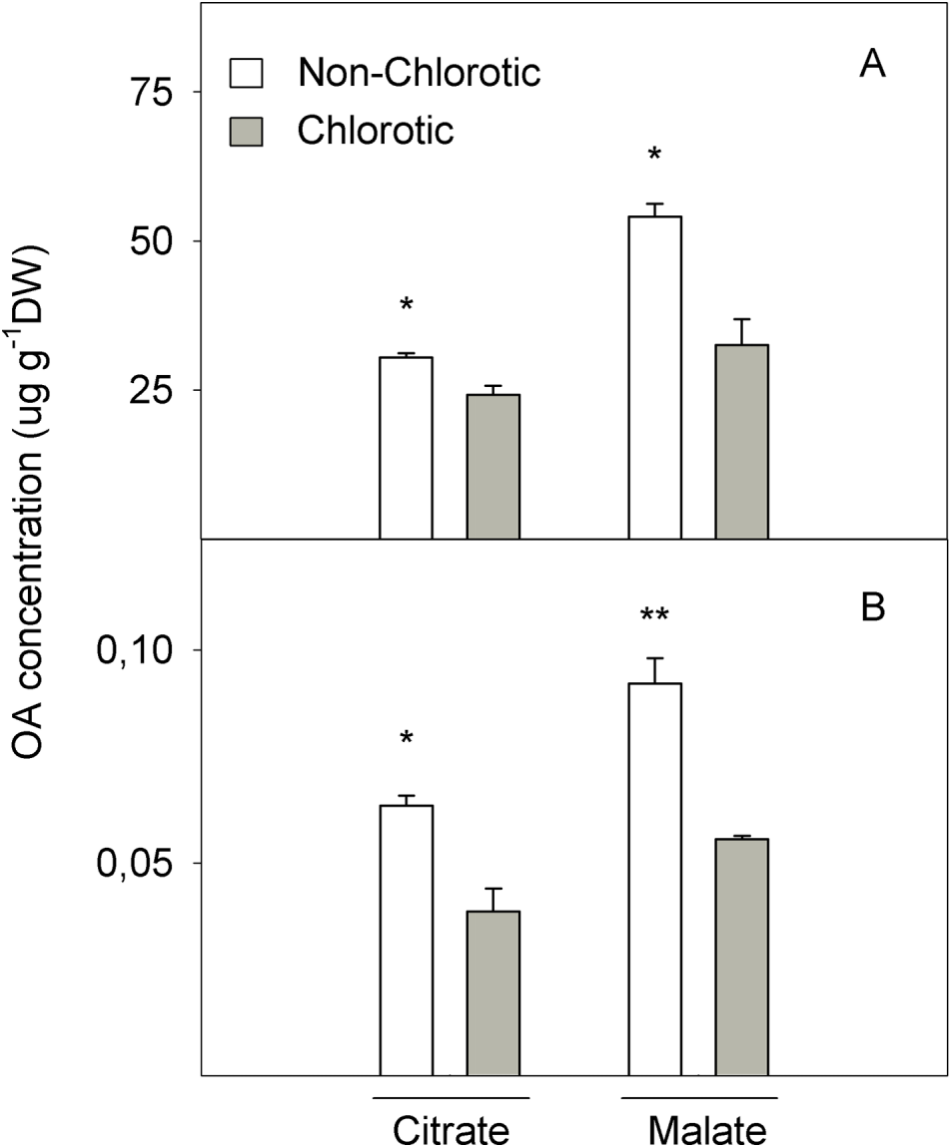
Organic acids (OA, citrate and malate) concentrations (in μg g^−1^ DW) determined in (A) the total tissue and (B) apoplast fraction of the non-chlorotic and chlorotic fully expanded leaves of ‘Navelina’ orange trees. Values are the mean ± SE of six biological replicates (*n* = 6). For a comparison of mean, a variance analysis (ANOVA), followed by the least significant differences (LSD) test, calculated at the 95% confidence level, was performed. Significant differences are indicated as follows: *p < 0.05; **p < 0.01. DW, dry weight.

Figure 7 shows the LMWOA concentration in the total leaf tissue and apoplast fraction. The major LMWOAs obtained in whole leaf homogenates were citrate and malate, and accounted for >90% of the total LMWOA pool (data not shown). In the total leaf tissue, citrate and malate concentrations were 25.7% and 124.3% higher in the non-chlorotic leaves than in the chlorotic ones. Malate was the major LMWOA in both samples, but the relation between malate and citrate was stronger in the non-chlorotic leaves than in the chlorotic ones (respectively 1.8- and 1.3-fold). Despite the accumulated quantity of LMWOA in the apoplast extract being much smaller than in the total leaf, it displayed a similar tendency to that described in the total organ tissue. Therefore, both citrate and malate concentrations were 65.0% higher in the leaves without Fe-deficiency symptoms than in the chlorotic ones.

## Discussion

Considerable research has been conducted into the influence of citrus rootstocks on tree growth, fruit quality and yield (Forner-Giner et al., 2003, 2011; Castle, Baldwin & Muraro, 2010; Yildirim et al., 2010; Da Silva et al., 2013) and scion behaviour is likely to depend, in part, on the rootstock-induced effects on leaf gas exchange, photosynthetic rates and photoassimilates (González-Mas et al., 2009; Jover et al., 2012). In our experiment, leaves from the same variety grafted on two different rootstocks presented different Fe-chlorosis symptoms (Fig. 1), but showed apparently similar total Fe levels on their leaves on the DW basis (Table 2). Initially, there could be three possible explanations for these differences:

(1) Inhibition of leaf growth: Despite observing no significant differences in total Fe when expressed on a DW basis, the marked reduction in both dry biomass and leaf area in the chlorotic leaves correlated with the low Fe concentration in the fully expanded leaves on an area basis (Table 2). As leaf growth was inhibited, consequently the high Fe concentration on a dry matter basis occurred due to the absence of Fe dilution. This phenomenon has also been related in grapevine, peach and pear leaves (Morales et al., 1998; Bavaresco, Giachino & Colla, 1999; Römheld, 2000). In addition, the high K^+^ concentration compared to Ca^2+^ in the chlorotic leaves compared with the green ones (Fig. 6) also likely suggested low carbohydrate synthesis, which slows down the movement of K^+^ from the leaf to the phloem vessels, and even stops it in chlorotic leaves and reduces biomass production (Hamzé et al., 1985). Inhibited leaf growth has been observed by other authors under field conditions, who have interestingly suggested that this fact could be of crucial importance in designing new ways of controlling Fe-chlorosis in fruit tree crops as Fe pools might be potentially capable of being remobilised with adequate agronomic activities, like spraying acid compounds (Morales et al., 1998; Rombolà et al., 2000).

However, the fact that the Fe concentration in chlorotic leaves fell within the range of normal levels for citrus crop (Reuther, 1973; Legaz et al., 2008) suggests that Fe could be immobilised somewhere in these leaves in an unavailable form. So in this case, the total Fe content may not be the best indicator of Fe-chlorosis. Interestingly, the green leaves presented a 2.5-fold higher active Fe concentration (Fe^2+^) than the chlorotic ones (Fig. 2), which positively correlated with the chlorophyll concentration and the plant’s photosynthetic state (Table 3), as previously reported in Medicago plants (M’sehli et al., 2014). The chlorotic leaves exhibited a marked reduction in the *Chl a* and *b* concentrations (60.7% and 48.2%, respectively) and SPAD value (62%). In contrast to the expected result of a high Chl a/b ratio in Fe-chlorotic leaves, the lower Chl a/b ratio in these leaves when compared with green ones, is likely linked to a marked Fe-deficient symptomatology as pointed by the strong decrease in SPAD value together with the sharp drop in leaf biomass both on DW and area bases. This tendency suggests the presence of chloroplasts with a poor development of grana, and therefore, severe chlorosis symptoms, as previously observed in other species (Stocking, 1975; Masoni, Ercoli & Mariotti, 1996). These authors stated that only when the deficiency symptoms were moderate to slight the Chl a/b ratio reached a maximum exceeding the level of green leaves. In addition, the A_CO2_ measurements and the maximum quantum yield of PSII (*F*_v_/*F*_m_) also lowered in the chlorotic leaves (Table 3). Similar results have been previously observed in different species submitted to Fe-chlorosis (Chouliaras et al., 2004; Pestana et al., 2005; Molassiotis et al., 2006; Jelali et al., 2011). As Fe^2+^ is fundamental in protoporphirine IX synthesis (the precursor of chlorophylls; Marschner, 1995), the low Fe^2+^ level in the chlorotic leaves (Fig. 2) was in accordance with the reduced chlorophyll concentration recorded in these leaves and, consequently, with the A_CO2_ value. Thus a drop in A_CO2_ has been related with the reduced activity of stroma enzymes, the number of thylakoids per granum, which lowers the photosynthetic electron transport rate, and the content of light-harvesting pigments (Chouliaras et al., 2005). The close relationship of Fe^2+^ with chlorophylls and chlorosis symptoms renders Fe^2+^ determination a better indicator of plant nutrient status (Katyal & Sharma, 1980).

(2) Fe inactivation in plants: The differences in chlorotic symptoms between both plants could also be explained by an alkalinisation process in leaf apoplast due to the elevated concentration of bicarbonate in soil solution, which would lower the level of active Fe freely available inside leaf tissues, similarly to what happens in roots. Despite this effect seems to be minor in our study as the pH in leaf apoplastic fluid did not significantly differ between both types of leaves (Fig. 6), the low decrease of total Fe in the cell sap from chlorotic leaves when compared with non-chlorotic ones (Fig. 2) suggests a high precipitation phenomenon of Fe not only in the apoplast but also in the cell sap. Accordingly, the supply of high HCO_3_^−^ concentrations in nutrient solution resulted in no or a slight increase in the pH of xylem sap or leaf apoplast fluid (Alhendawi et al., 1997; Nikolic & Römheld, 2002). Unlike these findings, Mengel, Planker & Hoffmann (1994) reported a considerable increase in the pH of the leaf apoplast in the supply to sunflower plants, although the experiment is not comparable with ours because pH was not measured in intact plants, but in excised leaves with immersion of leaf petioles in 10 mM of KHCO_3_.

In addition, no increase was observed in the total Fe amount found in the extracellular fraction in the chlorotic leaves, but a significant higher apoplast Fe in the non-chlorotic ones when expressed on an area basis. The absence of reduced Fe availability in the leaf apoplast has been observed in several species grown at high bicarbonate concentrations in soil solution (Nikolic & Römheld, 1999, 2002). These authors suggested that although some free HCO_3_^−^ could have remained in xylem sap following organic carbon fixation in roots, it was unlikely to cause a substantial increase in the pH in the leaf apoplast. So in agreement with them, Fe inactivation in the leaf apoplast is not likely to be a primary cause of the Fe-deficiency chlorosis induced by bicarbonate in citrus. Despite the notable difference in the total Fe concentration between roots and leaves, since the first one is the preferential organ for Fe accumulation in the plant (Martínez-Cuenca et al., 2013c), the second one is the main Fe sink and is where Fe participates in important plant-sustaining functions (Abadía, 1992). Therefore, Fe accumulation in the apoplast is also likely a key adaptive response for Fe-chlorosis tolerance in citrus leaves.

(3) Low Fe^3+^ reduction prior to its entry into the leaf symplast: The chlorotic leaves presented a marked reduction in FC-R activity and the *FRO2* gene expression level, which modulates Fe^3+^ to Fe^2+^ reduction and, therefore, the presence of Fe in the leaf in an active and available form. Accordingly, the Fe^2+^ concentration strongly correlated with enzyme behaviour (Figs. 2 and 4). Absence of FC-R activity induction, or even its decrease, has been previously described in leaves from other Fe-chlorotic plants (De la Guardia & Alcántara, 1996; Ojeda, Schaffer & Davies, 2005; Rombolà et al., 2000). It is assumed that FC-R activity is pH-dependent in roots and although its maximum activity occurs at around 5.5, it can even work at higher pH levels (Susin et al., 1996). In leaves, it actually exits a marked pH-dependence on both the Fe-source and light presence (De la Guardia & Alcántara, 1996; Rombolà et al., 2000; Larbi et al., 2001). However, no information is available in intact citrus leaf cells. In our study, we did not observe any differences in the apoplastic fluid pH between leaf samples, but FC-R activity was repressed in the chlorotic leaves compared to green leaves. So unlike roots, these results suggest that the pH in leaf apoplast is not an important factor involved in FC-R regulation at the leaf level in citrus grown in calcareous soils. In line with our results, no significant effect was observed between a varied pH of the incubation solution and the Fe^3+^ reduction and Fe uptake by leaf discs in sunflower and bean plants (Nikolic & Römheld, 1999), or the presence of HCO_3_ or nitrate in the soil solution but ammonium treatment lowered the apoplast pH and favoured Fe^3+^ reduction, which suggests its notable dependence on the type of anions supply (Mengel, Planker & Hoffmann, 1994). In the other hand, González-Vallejo et al. (2000) observed a marked decrease of FC-R activity beyond pH 6.0 in both Fe-sufficient and Fe-deficient plants, although the method is not comparable with ours as they used mesophyll protoplasts.

The second point of FC-R enzyme regulation could be the substrate source. We also analysed the LMWOA composition in the leaves and non-chlorotic samples, and recorded higher concentrations of citrate and malate acids than the chlorotic ones (Fig. 7). Interestingly, the malate concentration was the main organic acid recorded in both samples, but the relationship between malate and citrate was higher in the non-chlorotic leaves than in the chlorotic ones. The increased organic acids content in the non-chlorotic leaves, especially for the malate concentration, correlated with the greater FC-R activity and higher Fe^2+^ concentration in these leaves, which suggests that the FC-R enzyme is dependent not only on the amount of substrate, but also on the Fe source. Previous studies have concluded that: (1) according to Fe^3+^-malate and Fe^3+^-citrate spraying experiments, if Fe^3+^-malate can reach the plasma membrane of the leaf cell, it can provide a suitable source of Fe for FC-R and, therefore, for Fe uptake in leaves; (2) Fe^3+^-malate is characterised by having a greater ability to attract electrons compared to Fe^3+^-citrate; and (3) at a pH above 7.0, Fe^3+^-citrate is more unstable than Fe^3+^-malate, and turns into Fe hydroxyl citrate, which is less accessible for the reduction and uptake of the mesophyll cell wall at the plasma membrane (Bienfait & Scheffers, 1992; Rombolà et al., 2000).

Finally, the Fe^3+^ reduction rate also depends on the level of cytosolic NAD(P)H, so poor FC-R activity in chlorotic leaves could be impaired by the reduced availability of electron donors (Schmidt, 1999). This effect has also been described in the roots of citrus plants submitted to iron chlorosis and long-term waterlogging stresses (Martínez-Cuenca et al., 2015). Among the likely causes, the low level of photosynthetic products, such as triose phosphates in chlorotic leaves, which are transferred from chloroplasts to cytosol, involves the reduced generation of cytosolic reduction equivalents NAD(P)H (De la Guardia & Alcántara, 1996). Moreover, non-chlorotic leaves present enhanced POX and CAT activities compared to chlorotic leaves (Fig. 3), and both are antioxidant enzymes involved in H_2_O_2_ metabolism (Cakmak, 2000). Chlorotic leaves induce reactions to cope with cellular oxidative damage and ROS production which need to consume electron donors, so NADH availability for Fe-reduction activity in these leaves is restricted.

Therefore, according to our study, the influence of the rootstock on both FC-R activity and the amount of the active Fe form in the leaves seems to be the key factor to regulate Fe uptake in leaf cells. Despite the normal Fe total concentration in leaves, the enzyme impairment and the low Fe^2+^ level promote ‘Fe paradox’ phenomenon in citrus leaves, which, likely induce several responses in this organ to enhance Fe^3+^ availability, as occurs in the root system (Martínez-Cuenca et al., 2013a). First, the chlorotic leaves induced the expression level of the *HA1* gene (Fig. 5), which encodes the H^+^-ATPase enzyme involved in the H^+^ interchange between the cytoplasm and apoplast. To our knowledge, this is the first time that this acidification response has also been recorded in leaves with chlorosis symptoms to promote a favourable environment for the FC-R enzyme in this organ. Moreover, the high K^+^/Ca^2+^ ratio recorded in the chlorotic leaves (Fig. 6) also suggested increased H^+^ excretion across the plasma membrane by the H^+^-ATPase enzyme, and therefore promoted the acidification response, as previously described in other species (Marschner, 1995). Second, the chlorotic leaves also recorded a strong up-regulation of the *IRT1* gene, which encodes the major transporter responsible for high-affinity metal uptake in roots (Connolly, Fett & Guerinot, 2002; Vert et al., 2002, 2009; Martínez-Cuenca et al., 2013a, 2013c). The 2.8-fold reduction in the Fe^2+^ concentration inside cells in the chlorotic leaves likely enhanced *IRT1* gene expression by about 94% compared with the green leaves, which supports its key role in the response of citrus leaves to Fe-deprived conditions by enhancing Fe-transport capacity also in leaves, as in the root system (Martínez-Cuenca et al., 2013a). However, it was unable to improve Fe uptake inside the cell in this organ, as has also been described in citrus roots submitted to Fe-deficiency and flooding stresses (Martínez-Cuenca et al., 2015). This supports the idea that an increased uptake rate is the result of greater reduction capacity, and not of enhanced transporter activity as in the root system (Manthey, McCoy & Crowley, 1993; Martínez-Cuenca et al., 2013a, 2015).

In conclusion, (1) the rootstock is involved not only in Fe uptake by roots but also influences Fe acquisition system inside the leaf symplasm; (2) the increased sensitivity to Fe-deficiency as revealed by chlorosis and decreased biomass was correlated with lower FC-R activity and lower organic acid level in leaf cells, which could cause a decreased Fe mobility and trigger other Fe-stress responses in this organ to enhance acidification and Fe uptake inside cells; and (3) the chlorosis paradox phenomenon in citrus can occur occasionally as a combination of a marked FC-R activity impairment in the leaf and the strong growth inhibition in this organ.

## Supplemental Information

10.7717/peerj.3553/supp-1 Supplemental Information 1 Individual data.

## Abbreviations

ABTS: [2,2-azino-bis (3-ethylbenzothiazoline-6-sulphonic acid)]
CAT: Catalase (EC 1.16.1.7)
DW: Dry weight
EDTA: Ethylenediaminetetraacetic acid
FC-R: Ferric chelate reductase (EC 1.16.1.7)
FW: Fresh weight
H^+^-ATPase: Proton-ATPase
IRT: Iron transporter
LMWOA: Low-molecular-weight organic acids
POX: Peroxidase (EC 1.16.1.7)
PVF: Polyvinyl fluoride
PVPP: Poly(vinylpolypyrrolidone)
RT-PCR: Reverse transcription polymerase chain reaction

## Additional Information and Declarations

### Data Availability

The following information was supplied regarding data availability: The raw data has been supplied as Supplemental Dataset Files.
